# Lifespan and Fecundity Impacts of Reduced Insulin Signalling Can Be Directed by Mito‐Nuclear Epistasis in *Drosophila*

**DOI:** 10.1111/acel.70405

**Published:** 2026-02-04

**Authors:** Rita Ibrahim, Christin Froschauer, Susanne Broschk, David R. Sannino, Adam J. Dobson

**Affiliations:** ^1^ School of Molecular Biosciences University of Glasgow Glasgow UK; ^2^ Applied Zoology, Faculty of Biology Technische Universität Dresden Dresden Germany

## Abstract

The changing demography of human populations has motivated a search for interventions that promote healthy ageing, and especially for evolutionarily‐conserved mechanisms that can be studied in lab systems to generate hypotheses about function in humans. Reduced Insulin/IGF signalling (IIS) is a leading example, which can extend healthy lifespan in a range of animals, but whether benefits and costs of reduced IIS vary genetically within species is under‐studied. This information is critical for any putative translation. Here, in *Drosophila*, we test for genetic variation in lifespan response to a dominant‐negative form of the insulin receptor, along with a metric of fecundity to evaluate corollary fitness costs/benefits. We also partition genetic variation between DNA variants in the nucleus (nDNA) and mitochondrial DNA (mtDNA), in a fully‐factorial design that allows us to assess ‘mito‐nuclear’ epistasis. We show that reduced IIS can have either beneficial or detrimental effects on lifespan, depending on the combination of mtDNA and nDNA. This suggests that, while insulin signalling has a conserved effect on ageing among species, intraspecific effects can vary genetically, and the combination of mtDNA and nDNA can act as a gatekeeper.

## Graphical abstract

Insulin signalling extends lifespan across species but, in Drosophila, this effect can vary among populations. This variable response is underpinned by epistasis between mtDNA and nDNA.

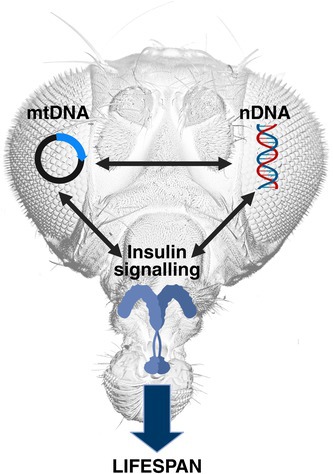

Human life expectancy has increased over recent centuries, increasing the prevalence of age‐related diseases such as cardiovascular disease, neurodegeneration and cancer (Singh et al. 2019; Niccoli and Partridge 2012; Li et al. 2021). This has motivated a search for fundamental mechanisms of ageing. The insulin/IGF signalling (IIS) pathway is a key regulator of ageing, from invertebrates to mammals (Ziv and Hu 2011; Kenyon et al. 1993; Clancy et al. 2001; Selman et al. 2008; Ikeya et al. 2009; Broughton et al. 2005; Dorman et al. 1995), and naturally occurring variants in this pathway are associated with human longevity (Singh et al. 2019; Chung et al. 2010). This evolutionarily‐conserved role has generated considerable interest in understanding mechanistically how and why IIS impacts ageing. However, IIS activity has adaptive functions early in life, for example, in transcription, translation, growth, metabolic regulation and mitogenesis (Li et al. 2014), and so any putative anti‐ageing intervention impairing the pathway must balance potential benefits against the probable costs to these traits. This necessitates understanding how costs and benefits vary, and what underpins this variation.

Ageing is subject to standing genetic variation (Yuan et al. 2020; Vaught et al. 2020; Durham et al. 2014), and consequently we expect individual variation in responses to anti‐ageing interventions. However, studies documenting such variation are scarce. We have shown genetic variation in *Drosophila* lifespan and mortality response to rapamycin (Ibrahim et al. 2024), and in multiple species variation in effects of dietary restriction has also been well documented (McCracken et al. 2020; Gautrey and Simons 2022; Durham et al. 2014; Green et al. 2022; Liao et al. 2013; Rikke et al. 2010; Francesco et al. 2024). With reference to IIS, mouse strains vary in their lifespan response to IGF‐1R mutation (Xu et al. 2014; Selman and Swindell 2018; Bokov et al. 2011), but otherwise we are not aware of systematic investigations, nor of candidate mechanisms. Characterising and understanding such variation is key to unlocking any putative translational potential of IIS impairment.

Accumulating evidence suggests that a significant amount of genetic variation is not caused by additive effects of variants, but rather by epistatic interactions between independent loci (Boyle et al. 2017). mtDNA and nDNA are inherited partly independently, because mtDNA is only inherited maternally, while nDNA is inherited from both parents: this generates ‘mito‐nuclear’ epistasis for diverse traits including lifespan, reproductive fitness, development, stress tolerance and metabolism (Garlovsky et al. 2025; Dowling and Wolff 2023). Here, we ask whether mito‐nuclear epistasis modulates response to IIS in female *Drosophila*, testing whether impacts on lifespan are universally positive or variable.

We impaired insulin signalling in *Drosophila* by ubiquitous expression of a dominant‐negative form of the insulin receptor *InR* (*InR* ^*DN*^), which has a well‐established lifespan‐extending effect, in studies performed predominantly in the *Dahomey* background (Ikeya et al. 2009; Slack et al. 2011; Dobson et al. 2019; Bolukbasi et al. 2017). We drove *InR* ^*DN*^ expression ubiquitously using the *Daughterless‐GeneSwitch* (*DaGS*) driver, activated by feeding on a chemical inducer, RU486. We established a new panel of fly lines to evaluate how the lifespan output of *InR* ^*DN*^ varies genetically, and how it can be shaped by variation in mtDNA, nDNA and mito‐nuclear epistasis. We refer to the various combinations of mtDNA and nDNA as mitonucleogenotypes (Dobson et al. 2023; Ibrahim et al. 2024). We confirmed that the stocks we used to found this panel were free of the cytoplasmic endosymbiont *Wolbachia*, which could confound our experiment by coinheritance with mtDNA (Figure S1A). We took flies from the *Canton‐S, Dahomey* and *w1118* backgrounds (henceforth C, D and E, respectively), and replicated a design that we and others have used previously to generate mitonuclear variation (Figure S1B) (Garlovsky et al. 2025; Vaught et al. 2020; Dobson et al. 2023; Ibrahim et al. 2024), introgressing nDNAs into mtDNA backgrounds by crossing males (nDNA donor) to females with the desired mtDNA. We iterated this procedure six times, which should eliminate > 95% of residual nDNA variation from F0 mothers. We used a fully‐factorial design, generating all nine possible combinations of mtDNA and nDNA (i.e., nine mitonucleogenotypes) in triplicated parallel backcrosses (i.e., 27 lines), into which we then crossed *DaGS* and *UAS‐InR* ^*DN*^ separately (i.e., generating 54 lines), from donors with matched nDNA (Figure S1B). We homozygosed the offspring and then crossed *DaGS* and *UAS‐InR* ^*DN*^ flies from the same lines, to finally generate nine mitonucleogenotypes, each triplicated to generate 27 lines altogether, all of which were heterozygous for each transgene and could therefore inducibly and ubiquitously express *InR* ^*DN*^. We fed RU486 (henceforth RU) to three‐day‐old adult females, inducing ubiquitous *InR* ^*DN*^ expression, then scored subsequent survival, and egg laying after one week of induction.

We compared survival in the presence/absence of RU for each mitonucleogenotype (Figure 1A), and analysed the data using parametric survival models (PSM). Overall, a three‐way interaction of RU, mtDNA and nDNA was apparent (LR ChiSq = 25.41, Df = 4, *p* < 0.0001). To identify what drove this interaction, we fit a second model which revealed mitonucleogenotype‐specific effects of RU (mitonucleogenotype:RU LR ChiSq = 101.54, Df = 8, *p* < 2.2e‐16). We used post hoc tests (estimated marginal means, henceforth EMmeans) to identify specifically how different combinations of mtDNA and nDNA led to distinct survival outcomes, both for the pool of three replicate populations (Figure S2A) and for each of the three constituent replicates per mitonucleogenotype (Figure S2B). We accepted the overall result in the analyses of the three pooled replicates only if the majority of replicate lines (i.e., two of three) were in agreement (Figure 1B). This approach gives a false discovery rate of 11%, that is, the probability (1/3) of randomly detecting one of the three possible results (i.e., lifespan increased, lifespan decreased, no effect), twice (1/3 × 1/3 = 0.11). This conservative methodology led us to reject a result from analysing the pool of *CE* populations (Figure S2A), because we were only able to detect an effect in population *CE3* (Figure S2B). Nevertheless, the overall result of the pooled analysis—a tripartite interaction between RU, mtDNA and nDNA—was robust to this filter. Specifically, the impact of RU was modulated by nDNA in the presence of mtDNAs *C* or *E*, shortening lifespan with nDNA *C* but extending lifespan with nDNA *D*; but mtDNA *D* blocked any response to RU regardless of nDNA (Figure 1A,B). Thus, the results indicated that (A) lifespan response to reduced insulin signalling can vary genetically, and (B) mito‐nuclear epistasis can play a role.

**FIGURE 1:**
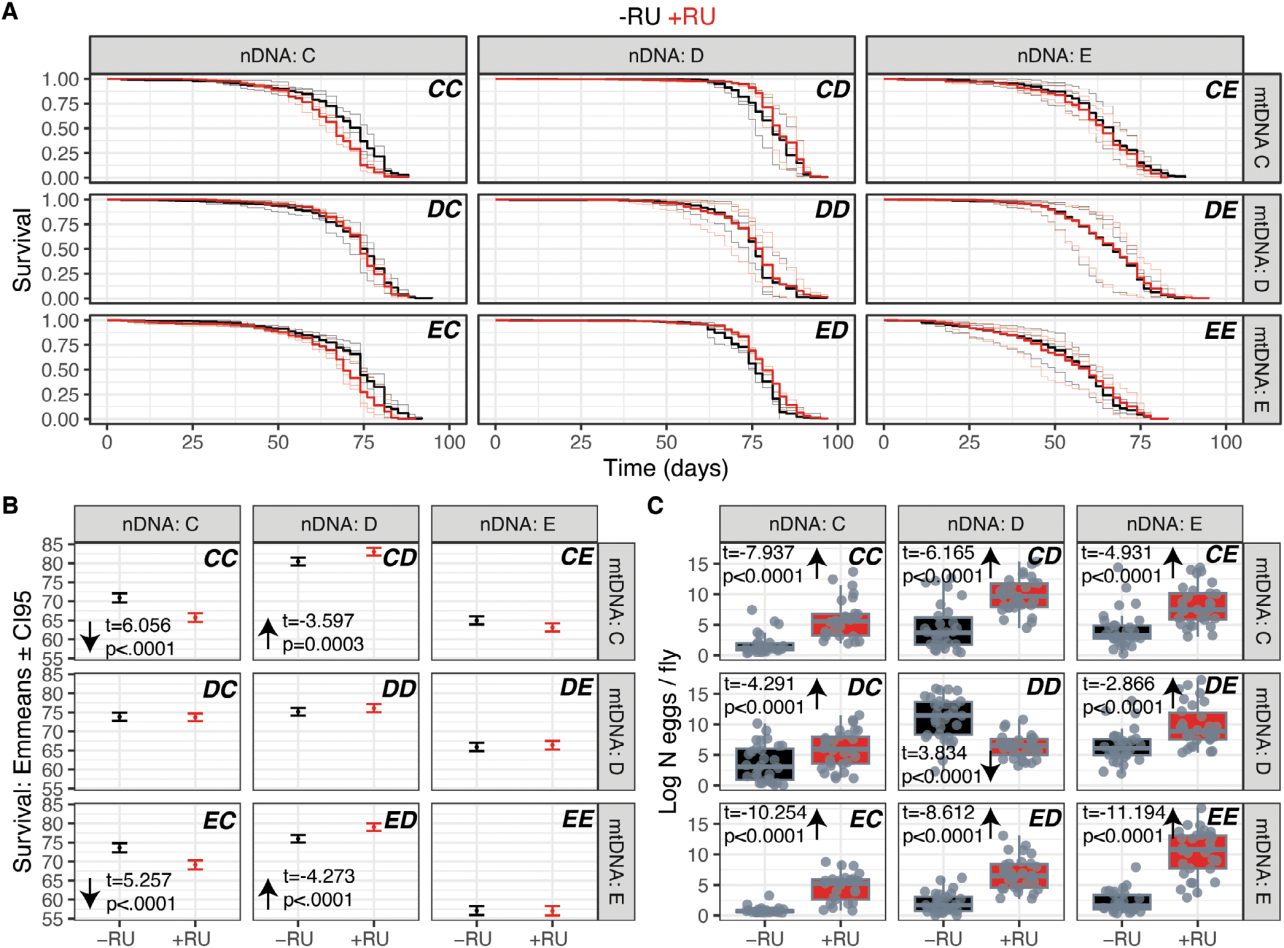
Mito‐nuclear epistasis directs the effect of reduced insulin signalling on lifespan and egg laying. Arrows on facets indicate effects of RU relative to control, when present. Lettering at top‐right of eachfacet indicates mitonucleogenotype. nDNA and mtDNA are indicated to top and right of each faceted panel, originating from ancestral backgrounds: *C* = *Canton S*, *D* = *Dahomey*, *E* = *w1118*. (A) Kaplan–Meier survival plots of *DaGS*/*UAS‐InR* ^*DN*^ flies, faceted by mitonucleogenotype. Survival of control flies shown in black, survival of flies fed 200 μM RU486 shown in red. Thin lines give survival of replicate populations, thicker lines give grand mean (pool) across replicates. Parametric survival model (PSM), mitonucleogenotype:RU LR ChiSq = 101.54, Df = 8, *p* < 2.2e‐16. (B) Post hoc analysis of RU486 effect per mitonucleogenotype. Each facet gives estimated marginal means (Emmeans) of the survival data plotted in panel A, with confidence intervals. Statistics from pairwise comparisons of effect of RU from PSM, stratified by mitonucleogenotype. Statistics are given only when significant effects were detected, negative t‐ratio indicates longer lifespan in RU‐fed flies than in controls. The analysis shows that nDNA can cause variation in lifespan response to *InR* ^*DN*^ induction, but this variation is blocked by mtDNA *D*. (C) Boxplots show eggs laid per female (nine days old), in the presence (red) or absence (black) of RU486. The boxplots represent the median, first and third quartiles and 5th and 95th percentiles. Statistics given on panels from post hoc tests on a negative binomial mixed model (mitonucleogenotype:RU Chisq = 180.601, df = 8, *p* < 2.2e‐16).

Lifespan‐extending interventions are expected to impair measures of fitness in early life, as a consequence of physiology deviating forcibly from optima that have been naturally selected to support fitness traits, such as reproduction. We therefore speculated that the variation we observed in lifespan response to InR^DN^ might negatively correlate a measure of reproduction. We measured fecundity early in life (in the same flies presented above in the survival assay) and, consistent with our expectation, in flies bearing mtDNA *D* and nDNA *D*, fecundity was decreased by RU. However, contrary to expectation, in every other mitonucleogenotype under study, fecundity was increased by RU (Figure 1B). These differences manifested as a mitonucleogenotype‐specific effect of RU (Negative binomial GLMM, mitonucleogenotype:RU Chisq = 180.601, df = 8, *p* < 2.2e‐16), and a significant RU:mtDNA:nDNA interaction (Negative Binomial GLM, ChiSq = 14.29, df = 4, *p* < 0.01). Thus, fecundity was increased both in flies that went on to be longer‐lived, shorter‐lived, or to exhibit no lifespan response upon *InR* ^*DN*^ induction. This suggests either that (A) the mechanisms that mediate lifespan response to *InR* ^*DN*^ either are not the same as those that determine fecundity response, and they can therefore exhibit independent patterns of genetic variation; or (B) whether lifespan and fecundity are determined by similar or distinct mechanisms depends on mito‐nuclear epistasis. In either case, our results suggest that fitness costs of *InR* ^*DN*^ expression are not obligate, and that in fact the relationship between lifespan cost and fitness benefit can be shaped by mito‐nuclear epistasis.

Impaired IIS clearly has capacity to promote healthy ageing, with effects reported widely across species (Partridge et al. 2010; Selman et al. 2008; Clancy et al. 2001; Kenyon 2010; Zhang et al. 2013; Murphy et al. 2003). It seems unlikely that beneficial effects could have been reported in species separated by hundreds of millions of years of evolutionary divergence if fundamental processes were not at play. Nevertheless, our present results, within just one species, suggest that one means of reducing IIS can have effects that vary within species. These results also complement our previous results, showing that the magnitude and timing of the effect of a pharmacological anti‐ageing intervention (rapamycin) are subject to mito‐nuclear variation (Ibrahim et al. 2024). Together, these studies imply that benefits of interventions with conserved effects are not necessarily universal, and that their benefits may be conditional. We manipulated genotype (specifically mitonucleogenotype), showing that different genotypes raised under standardised conditions exhibited different responses, but one intriguing possibility is that the capacity to benefit from reduced IIS may be subject to interactions between genotype and other variables, for example, environment, diet composition, timing of induction. It is possible that many genotypes have inherent capacity to benefit from anti‐ageing interventions, but context and conditions must be tailored appropriately. Such interactions could allow genotypes that experienced neutral or deleterious effects in the present study to show benefits in other contexts. Thus, the results imply that better understanding the context‐specificity of responses to anti‐ageing interventions should be a priority for the field. The basis for variation in response is likely complex, potentially incorporating interactions among genetics, nutrition, environment etc.; which will be difficult to disentangle. We therefore suggest that research should focus on characterising the emergent properties that predispose an individual to benefit from a given intervention.

We have shown specifically that the impact of impaired IIS is influenced by the ‘lock and key’ of mito‐nuclear epistasis. Emerging questions now are why should mtDNA, nDNA, and their epistatic interaction impact response to reduced IIS? Is this because IIS alters mitochondrial function in ways that depend on nDNA variants? Or do mito‐nuclear effects alter how IIS impacts cellular function? Mechanistic work is now required to address these issues. Furthermore, the present results complement and extend our previous finding that anti‐ageing impacts of rapamycin were more universally beneficial, but nevertheless shaped by mito‐nuclear epistasis. We therefore think it likely that other anti‐ageing interventions will be subject to genetic variation and likely mito‐nuclear variation.

We studied three commonly used lab backgrounds, including one (D, *Dahomey*) in which numerous studies have shown longevity upon *InR* ^*DN*^ induction. The *D* background requires the *Wolbachia* endosymbionts to derive lifespan benefit from *InR* ^*DN*^ (Ikeya et al. 2009). In this study, we ensured that ancestral populations were not infected by *Wolbachia*. We found that flies bearing *D* mitochondria blocked nDNA variation in response to *InR* ^*DN*^, however, *C* and *E* mitochondria were permissive. We have not addressed whether the block on variation imposed by *D* mitochondria is alleviated in the presence of *Wolbachia*, but this may be an interesting avenue of future symbiosis research, which may be relevant across *Wolbachia‐*hosting arthropods.

Altogether, our results provide new evidence that interventions that modulate ageing among species can nevertheless be subject to genetic variation within species, which can be underpinned by mtDNA variation, nDNA variation, and epistatic mito‐nuclear variation. Importantly, the intervention used here can lead to directionally different outcomes, implying that (1) we do not understand how genetic variation contextualises signalling, and (2) translation may require precise matching of interventions to individual biology.

## 1 Materials and Methods

Flies bearing the *w1118* mutation in the *CantonS*, ‘*w1118*’ and *OregonR* backgrounds were gifts from the lab of Julian Dow, University of Glasgow. Flies bearing the *w1118* mutation in the *Dahomey* background were gifts from the UCL Institute of Healthy Ageing. Flies bearing *DaGS* and *UAS‐InR* ^*DN*^ transgenes were gifts from Cathy Slack, University of Warwick. A panel of 9 mitonucleogenotypes was established as previously (Ibrahim et al. 2024; Dobson et al. 2023; Vaught et al. 2020) with 6 rounds of backcrossing, as described in Figure S1. Triplicating each mitonucleogenotype generated 27 populations altogether. Transgenes were backcrossed into the three respective nDNA backgrounds, crossed to mitonucleotypes bearing the same nDNA background, and homozygosed. Virgin females bearing *DaGS* (*n* = 45) from each of the 27 populations were crossed for 48 h in egg laying cages to males from the same populations bearing *UAS‐InR* ^*DN*^. Eggs were collected after an overnight egg lay, suspended in PBS, and 20 μL suspension was pipetted onto 60 mL sugar‐yeast‐agar (1×SYA) medium containing 10% (*w*/*v*) brewer's yeast (MP Biomedicals SR03010, LOT No. S4707), 1.5% (*w*/*v*) agar (Sigma A7002), 5% (*w*/*v*) sucrose (Fisher BP220‐10), 0.3% nipagin (10% in EtOH), 0.3% propionic acid (Bass et al. 2007). Upon eclosion as adults, flies were transferred to fresh food and allowed to mate 48 h before males were discarded. Females were fed food containing 200 μM RU486 (Cayman Chemical CAY10006317) to induce *InR* ^*DN*^ expression, with controls fed EtOH as a vehicle control. Survival was scored thrice weekly until all flies had died. Overnight egg laying was assayed after one week feeding on RU486.

Data were analysed in R 4.4.2. Survival data were analysed with a parametric survival model (rms::PSM). A logistic distribution was determined to best fit the data by comparing AIC values. Egg laying data were analysed with a negative binomial GLM (MASS::glm.nb). ANOVA tables were calculated with car::Anova, using Type‐3 tests. Post hoc tests were calculated with the EMMeans library.

## Author Contributions

R.I., C.F., S.B. and D.R.S. acquired data. R.I. and A.J.D. performed data analysis. A.J.D. conceived and supervised the study and wrote the manuscript.

## Funding

This work was supported by a UKRI Future Leaders Fellowship (Medical Research Council MR/S033939/1 and MR/Y019660/1, A.J.D.), the University of Glasgow (Lord Kelvin Adam Smith Scholarship, R.I.; Lord Kelvin Adam Smith Fellowship, A.J.D.) and Erasmus+ (S.B., C.F.).

## Conflicts of Interest

The authors declare no conflicts of interest.

## Supporting information


**Appendix S1:** acel70405‐sup‐0001‐AppendixS1.docx.

## Data Availability

The data that support the findings of this study are openly available at https://github.com/dobdobby/InR‐mito‐nuclear.
