# Supplementary material for: Lifespan and Fecundity Impacts of Reduced Insulin Signalling Can Be Directed by Mito‐Nuclear Epistasis in Drosophila
Source: Aging Cell. 2026 Feb 4;25(2):e70405. doi: 10.1111/acel.70405 (PMC12873451; doi:10.1111/acel.70405)
Supplement: Supplementary file 1 — Appendix S1: acel70405‐sup‐0001‐AppendixS1.docx. [file ACEL-25-e70405-s001.docx]

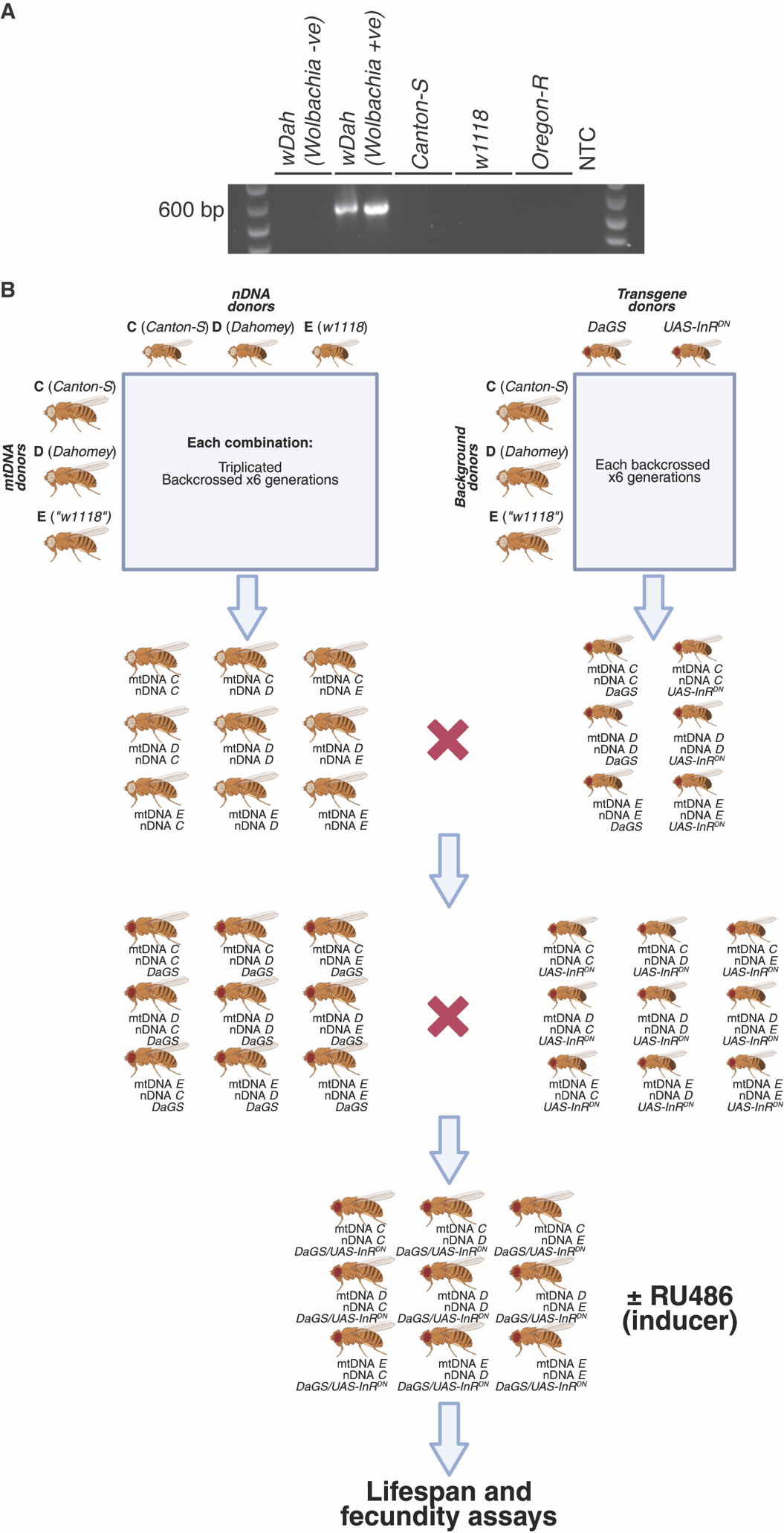


**Figure S1. Establishing new *Drosophila* populations to enable ubiquitous expression of dominant-negative *InR* in varied mitonucleogenotypes. A.** Lab populations were tested for presence of *Wolbachia* endosymbionts, by PCR using species-specific 16s primers. *Dahomey* flies known to be *Wolbachia* positive or negative (Ikeya et al. 2009) were included as positive and negative controls, respectively. *Canton-S*, *w1118* and *Oregon-R* populations in our lab were also found to be *Wolbachia*-negative. NTC=no-template control. **B.** 27 newly-established populations show mito-nuclear variation in egg laying. Populations were derived from *Canton-S (C), Wolbachia*-negative *Dahomey* *(D)*, and *w1118 (E),* by backcrossing nDNA over mtDNA (see panel C) in a fully-factorial design, generating nine mitonucleogenotypes, each in three replicate backcrosses. For each population the first letter represents mtDNA, second represents nDNA, and 1-3 represents independent replicate backcrosses (noting that 1-3 are independent across the nine mitonucleogenotypes), e.g. *CD1* = one replicate of mtDNA *C,* nDNA *D*. **C.** Combining functional genetics with mitonuclear variation. Nine mitonucleogenotypes were established (as shown in panels A and B). In parallel, *DaGS* and *UAS-InR^DN^* transgenes were backcrossed into corresponding ancestral *C, D* and *E* backgrounds. Transgene-bearing flies were then crossed to nDNA*-*matched mitonuclear flies (e.g. transgenes in nDNA *C* background were crossed to lines *CC1-3*, *DC1-3, EC1-3*; transgenes in nDNA *E* background were crossed to lines *CE1-3*, *DE1-3, EE1-3*, etc). Resulting heterozygotes were homozygosed, generating flies from nine mitonucleogenotypes bearing *DaGS* or *UAS-InR^DN^*, each in triplicate. Flies were crossed to finally generate nine mitonucleogenotypes (each triplicated) bearing *DaGS/UAS-InR^DN^.*


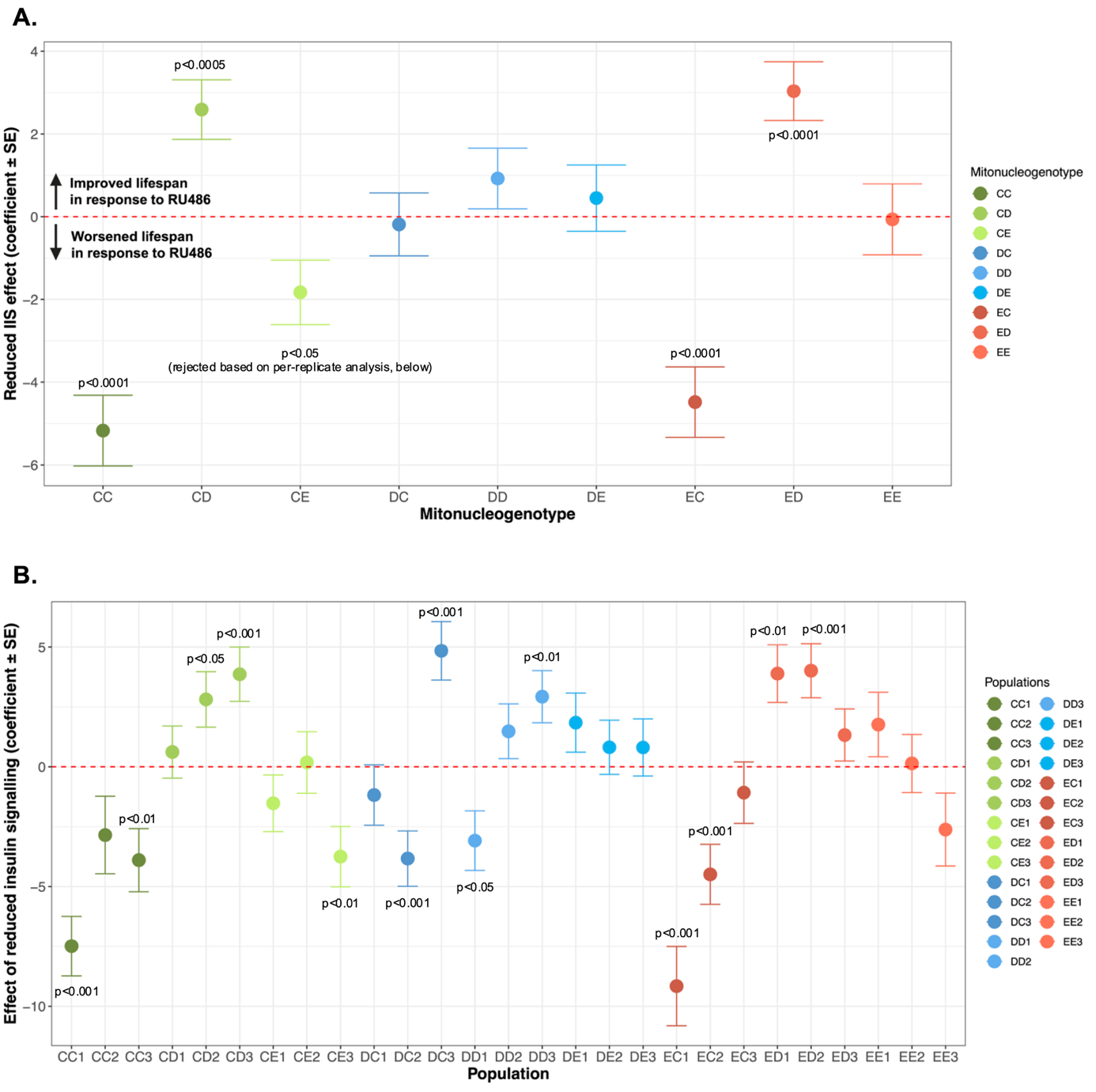


**Figure S2. Lifespan effects of InR^DN^ in nine mitonucleogenotypes and 27 constituent replicates.** **A.** Pooled populations - Coefficients from post-hoc analysis (impact of RU, stratified by replicate population), of EMMeans ±SE of a PSM (logistic distribution). **B.** Constituent replicates of the pooled populations - Coefficients from post-hoc analysis (impact of RU, stratified by mtDNA and nDNA), of EMMeans ± SE. Coefficients reflect the impact of InR^DN^ induction on lifespan. Coefficients are multiplied -1*x, such that higher values correspond to extended lifespan. Red horizontal represents no impact of InR^DN^.
